# Mental health among children of mothers with multiple sclerosis: A Danish cohort and register‐based study

**DOI:** 10.1002/brb3.1098

**Published:** 2018-09-21

**Authors:** Johanna Balslev Andersen, Julie Yoon Moberg, Janni Niclasen, Bjarne Laursen, Melinda Magyari

**Affiliations:** ^1^ Danish Multiple Sclerosis Registry, Department of Neurology Copenhagen University Hospital, Rigshospitalet Copenhagen Denmark; ^2^ Independent Psychologist; ^3^ National Institute of Public Health University of Southern Denmark Copenhagen Denmark; ^4^ Danish Multiple Sclerosis Center, Department of Neurology Copenhagen University Hospital, Rigshospitalet Copenhagen Denmark

**Keywords:** children, Danish National Birth Cohort, mental health, multiple sclerosis, strengths and difficulties questionnaire

## Abstract

**Background:**

Multiple sclerosis is associated with an increased risk of developing physical, cognitive, and mental health problems. Current studies have demonstrated variating outcomes of parental multiple sclerosis mental health problems and their children's mental health development.

**Objective:**

The purpose of this study was to investigate whether maternal multiple sclerosis is associated with the mental health status of their child.

**Methods:**

Data from the Danish National Birth Cohort (DNBC) were merged with information from the Danish Multiple Sclerosis Registry. Two proxies, total difficulties score and prediction of any psychiatric diagnosis based on the strengths and difficulties questionnaire, were used to measure the mental health status of the children. The two groups were compared using Mann–Whitney and logistic regression analyses.

**Results:**

For the total difficulties score the control and exposed group consisted of respectively *n* = 42,016 and *n* = 40, and for the prediction of any psychiatric diagnosis respectively *n* = 16,829 and *n* = 17. We found no statistically significant association between maternal multiple sclerosis and mental health status on neither of the proxies.

**Conclusion:**

Maternal multiple sclerosis did not show any association with the mental health status of their children at age eleven. On the contrary, other studies conclude that there is an association between maternal multiple sclerosis and the child's mental health status, one especially mediated by the maternal mental health status.

## 1 INTRODUCTION

Multiple sclerosis (MS) causes a wide array of debilitating health effects resulting in physical and cognitive impairment (MS Symptoms, n.d.). Common cognitive impairments include memory loss, problems with processing of information, organizing, problem solving, keeping focus, and attention. Depression and anxiety are the most frequent comorbidities in persons with MS (Boeschoten et al., 2017).

Chronic illness in adults is associated with an increased risk of developing mental health problems (Pakenham & Cox, 2014; Sieh, Meijer, Oort, Visser‐Meily, & Van der Leij, 2010). Furthermore, offspring of mothers with a chronic disease have been reported to display an increased risk of externalizing problems (Sieh et al., 2010).

Parental impact on the early childhood and beyond affects various aspects of children's lives including social skills, emotional regulation, self‐control, mental health, and brain development (Sanders, Kirby, Tellegen, & Day, 2014). In summary, good mental health has a positive influence on adulthood such as effective economic participation as an adult, good physical and mental health, and productive social relations.

The literature on the impact of maternal MS on children is scarce and diverse. Some studies indicate an adverse impact including psychosocial problems (Bogosian, Moss‐Morris, & Hadwin, 2010), emotional immaturity, vulnerability (Razaz, Joseph, et al., 2016), social competences (Razaz, Nourian, Marrie, Boyce, & Tremlett, 2014), and an increased risk of mood or anxiety problems (Razaz, Tremlett, et al., 2016). A recent study using the strengths and difficulties questionnaire (SDQ) to measure psychosocial development confirms the association between mental health problems of parents with MS and increased internalizing and externalizing symptoms affecting their children (Bogosian, Hadwin, Hankins, & Moss‐Morris, 2016). Other studies find no adverse association of parental MS with children's development outcomes (Razaz et al., 2015) and no difference in overall difficulties between children of parents with MS and the general community (Steck et al., 2007), and some studies have shown an increase in prosocial behavior (Pakenham & Cox, 2012) and an increased feeling of being more empathic and grown‐up than peers (Bogosian, Moss‐Morris, Bishop, & Hadwin, 2011).

The purpose of this study was to investigate whether maternal MS is associated with the mental health status of their child at age 11.

## 2 METHODS AND MATERIALS

### 2.1 Cohorts

Two subgroups were selected from the Danish National Birth Cohort, a nationwide birth cohort (Om BSIG ‐ Statens Serum Institut, n.d.): 1. the exposed group, that is, the “MS Offspring Cohort,” including children at age 11 with mothers diagnosed with MS, and 2. “the reference group,” that is, children at age 11 of mothers without MS.

#### 2.1.1 The Danish Multiple Sclerosis Registry (DMSR)

The DMSR is a nationwide population‐based registry which was established in 1956 (Koch‐Henriksen, Magyari, & Laursen, 2015). DMSR consists of all registered MS cases with a disease onset after 1947. Data on mothers’ MS diagnosis according to the time perspective diagnostic criteria were retrieved from the DMSR.

#### 2.1.2 Danish National Birth Cohort (DNBC)

All women in Denmark who became pregnant from April 1996 to October 2002 were eligible to participate in the nationwide longitudinal epidemiological study DNBC (Om BSIG ‐ Statens Serum Institut, n.d.). A total of 100,419 women consented to participate. The purpose of establishing this cohort was to assemble a large database about prenatal and early‐life exposures, as well as various contextual data such as lifestyle choices, nutrition, socioeconomic factors, and emotional and mental health.

Participants were invited twice during their pregnancy, when their child was 6 and 18 months of age and finally when the child was 7, 11, and 14 years of age. At age 11, both parents and children were invited to fill in a questionnaire investigating the health and well‐being of the child, their social relations, school abilities, development, physical activity, diseases, medication, smoking and alcohol, use of cell phones, etc. In addition, these questionnaires also included the strengths and difficulties questionnaire (SDQ), which is a 25‐item screening tool to investigate the mental health status of the children which both the child, mother, and the child's teacher were invited to answer. The questionnaire at age 11 was only available online. Link and password were sent to the home address of the child, one combination for the child and one for the mother. The participatory invitation letter encouraged the parent to let the child answer the questionnaire on its own, although it was not registered whether the child had help filling it out. A “quick‐answer” incentive (4 weeks from receiving the invitation to participate) was used to increase the response rate. Reminder letters were sent twice with 4‐week gap if not completing the online questionnaire after receiving the participatory invitation letter.

Data from the DMSR and the DNBC were merged using the unique Danish personal identification number (CPR). The Danish CPR number is given to all Danish citizens at birth and can be used to link cohorts, databases, and registries.

### 2.2 Samples

The eligible sample of children in the DNBC at age 11 was *n* = 68,559.

**Figure 1:**
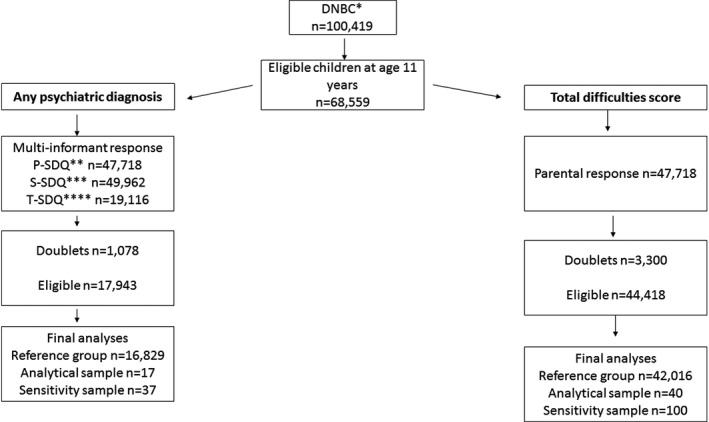
Flowchart presenting participation in DNBC and in this study. *Danish National Birth Cohort. **Parental participation rate at child age 11 years. ***Self‐reported participation rate at child age 11 years. ****Teacher participation rate at child age 11 years

Two subsamples, an analytical sample and a sample for sensitivity analyses, were compiled according to the outcome proxies, namely total difficulties score based on parental response and prediction of any psychiatric diagnosis based on multi‐informant response (parent, teacher, and child itself) (Figure 1).

**Figure 2:**
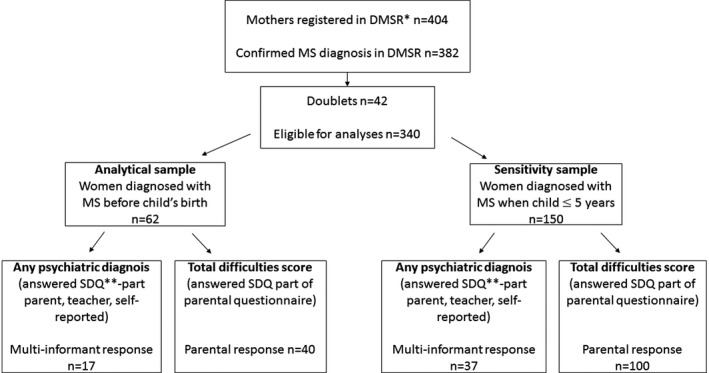
Flowchart presenting the distribution and participation of the exposed group in this study. *The Danish Multiple Sclerosis Registry. **The Strength and Difficulties Questionnaire

From the exposed group (where the mother had been diagnosed with MS before the child's birth), a total of 62 children were eligible for the analytical sample. For the sensitivity analyses (where mothers had to be diagnosed with MS before the child turned 6 years), a total of 150 children were eligible (Figure 2).

The exclusion criterion was to be a sibling and/or twin as only one child per mother could participate in this study. This applied to both the reference group and the exposed group to avoid clustering, and children were randomly selected from both groups.

### 2.3 Instruments

#### 2.3.1 The Strengths and Difficulties Questionnaire (SDQ)

Mental health was measured by means of the SDQ. The SDQ is a brief behavioral and emotional questionnaire based on nosological concepts, which screens for psychological difficulties in children (Goodman, 1997). Its scores have been used as a proxy for mental health. An extended version of the SDQ was developed based on the fact that symptoms alone were not accurate enough as a guideline for a psychiatric diagnosis (Goodman, 1999).

The SDQ contains 25 items (SDQ info, n.d.), which are grouped into five subscales: hyperactivity, emotional, conduct, peer relationship, and prosocial, each consisting of five items. The total difficulties score is obtained from adding the scores from the former four problem scales. The latter is not incorporated into the total difficulties score, as prosocial behavior is not deemed relevant for predicting psychological difficulties (Goodman, 1997).

The total difficulties score was classified as “normal,” “borderline,” and “abnormal” according to previous UK and Danish validation studies (Goodman, 1997; SDQ info, n.d.).

The probability of any psychiatric diagnosis was based on a predictive algorithm that includes the 20 items from the SDQ described above and the items from the extended version regarding the impact burden and chronicity of the symptoms (Goodman, 1999).

Social status variable is a composite variable based on the classification system used by Statistics Denmark (Find statistics, n.d.). The variables included are “working skills,” “working functions,” and “educational accomplishment” as defined by the International Standard Classification of Education.

### 2.4 Statistical analysis

The data were analyzed using SAS Enterprise Guide 7.13. Nonparametric tests were used as data were not normally distributed.

The total difficulties score was grouped based on the cutoff values obtained from the Danish SDQ validation (“normal,” “borderline,” “abnormal”) (Niclasen et al., 2012), while the predictive algorithm divides its results into three groups for having any psychiatric disease—“unlikely,” “possible,” and “probable.”.

Comparisons of associations were performed with chi‐square test and statistical analyses using two‐tailed Mann–Whitney *U* test. Both tests were performed on raw total difficulties score and stratified for sex. All tests and comparisons have been performed on both the analytical sample and the sensitivity sample.

This study had two outcomes as proxies for mental health: the total difficulties score based on parental ratings and the predictive algorithm for any psychiatric diagnosis, based on multi‐informant rating. Multivariate logistic regression analyses were performed to adjust for the sex of the child and social status of the mother. To perform multivariate logistic regression, dichotomous variables were created by merging the “normal” and “borderline” category within the total difficulties score and the “unlikely” and “possible” categories within any psychiatric diagnosis.

### 2.5 Ethics

The DNBC is approved by the Committee on Biomedical Research Ethics reference number (KF) 01‐471/94. Until September 2015, the cohort was approved by the Danish Data Protection Agency for research and statistical use (j. 2012‐54‐0268). From September 2015 and ongoing, the research and statistics are approved by Statens Serum Institut, which is a subsidiary of the Danish Ministry of Health.

All participants in the DNBC signed an informed consent form prior to cohort inclusion. Due to the nonintervention nature of the cohort study, approval from the Danish Ethical Committee was not required.

The CPR was encrypted to ensure anonymization.

## 3 RESULTS

The control group for total difficulties score consisted of 42,016 individuals and 16,829 for any psychiatric diagnosis (Figure 1, Tables 1 and 2). The exposed group for total difficulties score consisted of 40 children of mothers diagnosed with MS in the analytical sample and 100 children in the sensitivity sample. The exposed group for any psychiatric diagnoses consisted of 17 children in the analytical sample and 37 in the sensitivity sample (Figure 2). To be included in the analyses, a completed SDQ was required from child, parent, and teacher.

**Table 1:** Baseline description of mothers with and without MS in this study. The social status is based on years of education and work skills

	Mothers with MS	Mothers without MS
*n*	382	68,177
Mother's age at child's birth, mean (range)	29.7 (16–46)	30.1 (16–47)
Mother's age at MS diagnosis (range)	35.5 (16–55)	*N*/A
Mother's social status		
1. Higher‐grade professionals	32	5,781
2. Lower‐grade professionals	76	17,502
3. Skilled work	81	11,073
4. Unskilled work	92	14,214
5. Studying	55	8,010
6. Outside the workforce	33	2,686
7. Not specified	4	555

**Table 2:** Baseline description of children with mothers with and without MS

	MS offspring	Reference cohort
*n*	382	68,177
Sex, (%)		
Boys	48.5%	50.3%
Girls	51.5%	49.7%
Year of birth, median (range)	2000 (1997–2003)	2000 (1996–2003)
Distribution of children based on when the mother got diagnosed with MS, (%)		
Before birth	18,2%	N/A
0–5 years	25,8%	N/A
>5 years	56%	N/A

The analytical sample only included children of mothers, who had been diagnosed with MS before childbirth, whereas the sensitivity analyses included children of mothers who had been diagnosed with MS before the child had turned 6 years.

### 3.1 Analytical sample

The results of the raw parental total difficulties score of children of mothers diagnosed with MS and the control group were comparable, but without statistical significance. When stratified for sex, the results remained without statistical significance (Table 3).

**Table 3:** Mann–Whitney *U* test displaying the significance of MS in the analytical sample when comparing any psychiatric diagnosis, the parental rating of the raw total difficulties score and stratified for sex

	Reference children, *n*	MS offspring Analytical sample (mother diagnosed with MS before child's birth), *n*	*p*‐value*
SDQ parent at age 11 years	42,016	40	0.95
Parental rating—only boys age 11	20,851	13	0.94
Parental rating—only girls age 11	21,165	27	0.73
Any psychiatric diagnosis	16,829	17	0.18

*p‐value set to significant at <0.05.

The distribution of any psychiatric diagnosis was also statistically insignificant between children of mothers diagnosed with MS and the control group.

We performed bivariate and multivariate logistic regression analyses adjusting for the child's gender and the social status of the mother and found that maternal MS did not influence the risk of psychiatric diagnosis neither when comparing “abnormal” versus “borderline+normal” derived from the total difficulties score nor when comparing “probable” versus “possible+unlikely” derived from the prediction of having any psychiatric diagnosis (Table 4).

**Table 4:** Logistic regression analyses of the significance of MS for any psychiatric diagnosis and total difficulties score for the analytical sample, also when adjusted for child's gender and social status of the mother

	Reference children	Unadjusted analyses MS offspring Analytical sample (mother diagnosed with MS before child's birth)	Adjusted analyses MS offspring Analytical sample (mother diagnosed with MS before child's birth)
		*p*‐value* OR (CI 95%)	*p*‐value* OR (CI 95%)
*n*	42,016	40	40
Parental SDQ at child age 11 years			
Total difficulties score		0.66	0.81
Abnormal versus Borderline+normal		0.84 (0.38;1.82)	0.91 (0.41;1.98)
*n*	16,829	17	17
Any psychiatric diagnosis		0.36	0.36
Probable versus Possible+unlikely		0.62 (0.22;1.72)	0.63 (0.21;1.72)

*p‐value set to significant at <0.05.

### 3.2 Sensitivity analyses

The sensitivity analyses were similarly performed on total difficulties score and any psychiatric diagnosis. No statistically significant differences were found.

## 4 DISCUSSION

In this study, we investigated whether maternal MS affects the mental health status of their child at age 11. We found no statistically significant differences on neither of the proxies used for the mental health status of the child, namely total difficulties score and having any psychiatric diagnosis when comparing the exposed and control groups.

The use of the SDQ has some limitations as its primary outcomes are based on a self‐reported questionnaire. In general, self‐reported questionnaires induce recall bias, although this would be assumed to be equally distributed among the exposed and unexposed subjects. At age 11, both children and adults had to complete a questionnaire each in which the individuals were asked to relate to “the last 6 months” with regard to all 25 items. The accuracy of correctly recalling the occurrence of events in the past six months can be questioned for both adults and children. Furthermore, caution should be applied to the perception of abstract time‐related questions and literacy skills at age 11. The outcomes were compared to the Danish normative data for, respectively, boys and girls (Niclasen et al., 2012; SDQ info, n.d.). The SDQ is one of the few valid and internally used screening tools for psychiatric diagnosis in children, but as a screening tool, it could not be sensitive enough to detect potential associations.

We found no statistical differences in the mental health status between MS offspring and children of mothers without MS at age 11. Previous research is scarce and includes both quantitative and qualitative studies of varying quality with diverse conclusions (Razaz et al., 2014), which indicate the challenges of capturing the impact of parental MS on children's psychosocial development and well‐being. Recent nationwide Danish population‐based registry studies found that MS offspring achieved statistically significant higher average grades when finishing basic school (Moberg et al., 2016), but the employment rate at age 30 among MS offspring was lower than that within the reference group, and MS offspring had an 31% increased risk of receiving disability pension at age 30 (Moberg et al., 2017).

Razaz et al. (2016) examined developmental health outcomes of MS offspring at age 5 years and found no adverse association of parental MS with children's developmental outcomes. However, they did report that children of MS parents with mental health problems were associated with higher risks of developing vulnerability on the social competences and emotional maturity.

Several studies indicate parental mental health, emotional distress, and expressed emotions are more important determinants for the impact of MS on their offspring's psychosocial development (Bogosian et al., 2016; Razaz et al., 2014, 2015 ; Razaz, Joseph, et al., 2016) than the severity and type of MS. Therefore, the lack of information on mental health problems of the mother is a limitation of this study.

We cannot exclude that nonparticipation bias in the DNBC affected our results.

Two nationwide Danish register‐based studies have investigated nonparticipation rates and loss to follow‐up in the DNBC. Greene, Greenland, Olsen, and Nohr (2011) investigated loss to follow‐up at age 7 in the DNBC and concluded that participants lost to follow‐up were slightly younger, more overweight prior to pregnancy, more often smokers (and heavier smokers) during pregnancy, and belonging to the lower socio‐occupational group. Greene et al. (2011) compared the percentage of participants in the low, middle, and high socio‐occupational groups at baseline and follow‐up and concluded that those lost to follow‐up were almost equally distributed between the three groups in both the baseline and follow‐up groups.

Several studies demonstrate an association between low social status and mental health problems (Allen, Balfour, Bell, & Marmot, 2014; Center on The Developing Child, 2012; WHO, 2013).

Jacobsen, Nohr, and Frydenberg (2010) investigated the socioeconomic nonparticipation bias of women in the DNBC compared to the background population. They demonstrated an underrepresentation of 18% if unemployed, 62% if outside the workforce or with no further education than compulsory school, 22% if in the lowest income group, and 38% if they received a high proportion of social benefits. This shows a clear difference in the social gradient of participation in the DNBC.

Madsen, Hohwü, Zhu, Olsen, and Obel (2017) investigated the relative representation of childhood psychiatric diagnoses in the DNBC compared to the general population and found an underrepresentation of the prevalence of childhood psychiatric diagnoses in the DNBC compared with the general population. This indicates that the difference in social gradient of participation in the DNBC presented by Jacobsen et al. (2010) does affect the representativeness of the DNBC cohort compared to the general population. The level of education and occupation appeared to be the strongest predictors for participation, with the highest participation rates among healthcare professionals and lowest participation rates among those with no or unknown education.

Using data from a nationwide population‐based MS registry with a high completeness linked to a national birth cohort is a strength of this study, despite the fact that the DNBC has an increased representation of women with a higher socioeconomic background and a lower representation of childhood psychiatric diagnoses compared to the background population. The differences between the background population and the DNBC could adversely affect the possibility of finding an association between maternal MS and the child's mental health status.

To ensure the maximum effect time of maternal MS on the child's development, the criteria for MS diagnosis before the child's birth were chosen which reduced the analytical sample to 62. Likewise, the criteria for the sensitivity sample were chosen based on the child being affected from maternal MS more than half of their lifetime. Furthermore, to perform regression analyses, variables were recategorized from trichotomies to dichotomies that may have caused an increased number of “false negatives,” that is, children who are borderline when dichotomizing (Goodman, Ford, Corbin, & Meltzer, 2004; Goodman, Renfrew, & Mullick, 2000).

## 5 CONCLUSION

We could not show an association between maternal MS and children's mental health status at age 11. One should consider mental health‐related problems often develop over time, and therefore, repeating the study after some years may answer some of the unanswered questions.

## CONFLICT Of INTEREST

No conflict of interests are noted for either of the authors.
